# Attenuation of Na/K-ATPase Mediated Oxidant Amplification with pNaKtide Ameliorates Experimental Uremic Cardiomyopathy

**DOI:** 10.1038/srep34592

**Published:** 2016-10-04

**Authors:** Jiang Liu, Jiang Tian, Muhammad Chaudhry, Kyle Maxwell, Yanling Yan, Xiaoliang Wang, Preeya T. Shah, Asad A. Khawaja, Rebecca Martin, Tylor J. Robinette, Adee El-Hamdani, Michael W. Dodrill, Komal Sodhi, Christopher A. Drummond, Steven T. Haller, David J. Kennedy, Nader G. Abraham, Zijian Xie, Joseph I. Shapiro

**Affiliations:** 1 Marshall University, Joan C. Edwards School of Medicine, 1600 Medical Center Drive Huntington, 25701 United States; 2 University of Toledo, College of Medicine, 3000 Arlington Ave. Toledo, OH 43614 United States; 3 New York Medical College, 15 Dana Road, Valhalla, NY 10595-1554 United States.

## Abstract

We have previously reported that the sodium potassium adenosine triphosphatase (Na/K-ATPase) can effect the amplification of reactive oxygen species. In this study, we examined whether attenuation of oxidant stress by antagonism of Na/K-ATPase oxidant amplification might ameliorate experimental uremic cardiomyopathy induced by partial nephrectomy (PNx). PNx induced the development of cardiac morphological and biochemical changes consistent with human uremic cardiomyopathy. Both inhibition of Na/K-ATPase oxidant amplification with pNaKtide and induction of heme oxygenase-1 (HO-1) with cobalt protoporphyrin (CoPP) markedly attenuated the development of phenotypical features of uremic cardiomyopathy. In a reversal study, administration of pNaKtide after the induction of uremic cardiomyopathy reversed many of the phenotypical features. Attenuation of Na/K-ATPase oxidant amplification may be a potential strategy for clinical therapy of this disorder.

We have shown that the plasmalemmal Na/K-ATPase has a signaling function in addition to and distinct from its pumping function^1^,^2^,^3^. We have also demonstrated that this signaling function may amplify oxidants and increase cellular oxidant stress; conversely the blockage of this signal cascade with a designed peptide, pNaKtide, may attenuate oxidant stress^4^,^5^,^6^,^7^. In particular, we have recently shown that pNaKtide antagonizes the cellular generation of reactive oxygen species (ROS) in response to several stimuli in a dose-dependent manner both *in vitro* and *in vivo* in models of adipogenesis and obesity^4^. Alternatively, the induction of HO-1 with a variety of agents has also been shown to attenuate oxidant stress^8^,^9^,^10^,^11^. Patients with chronic kidney disease are at great risk for cardiovascular disease events and mortality and develop the clinical phenotype called “uremic cardiomyopathy”^12^,^13^. As oxidant stress is a constant feature of both clinical^14^ and experimental uremic cardiomyopathy^15^, we reasoned that increased detoxification of oxidants by HO-1 induction as well as attenuation of Na/K-ATPase signaling mediated oxidant amplification with pNaKtide might ameliorate the phenotypical changes in experimental uremic cardiomyopathy.

## Results

### Effect of telecinobufagin (TCB) and pNaKtide on collagen production and signaling in C57BL/6 mouse primary cardiac fibroblast cells

**Figure 1:**
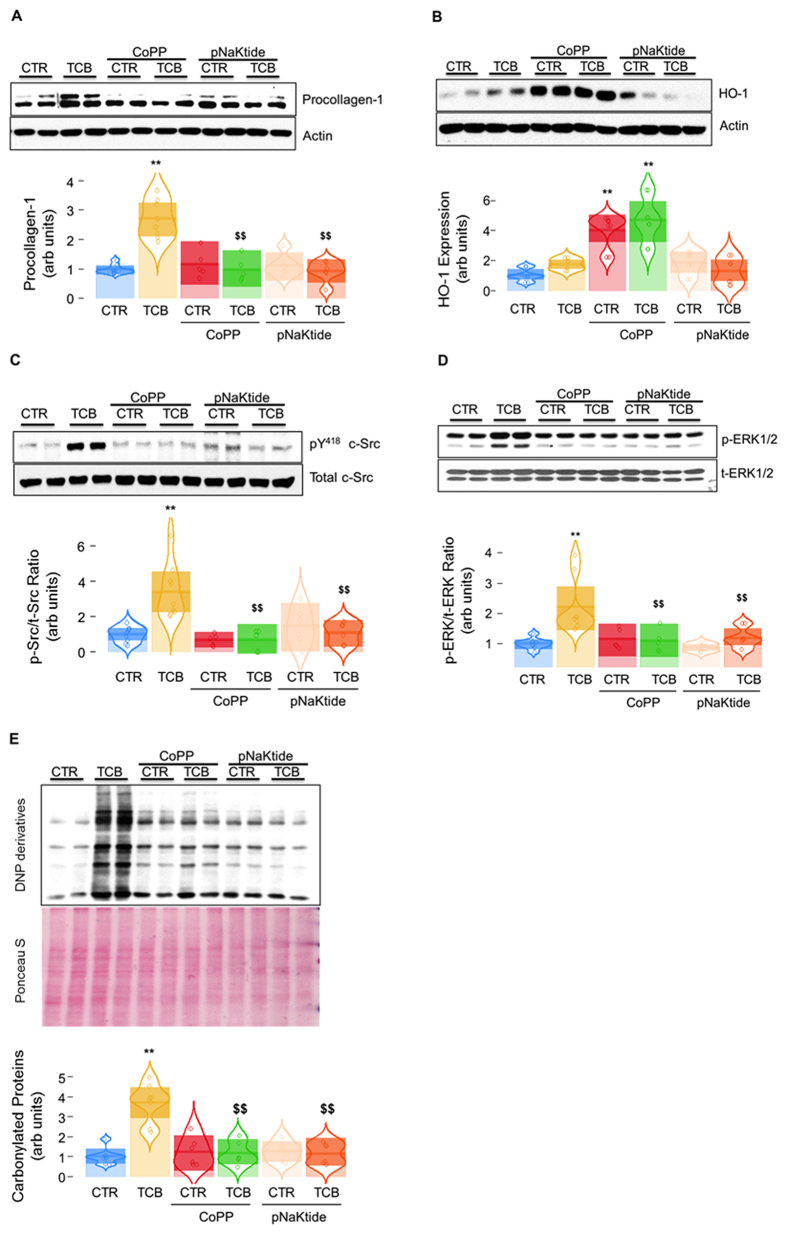
Induction of HO-1 with CoPP and blockade of Na/K-ATPase signaling with pNaKtide ameliorated the effect of TCB in murine cardiac fibroblasts. Primary culture of C57BL/6 mouse cardiac fibroblasts were used for these *in vitro* studies demonstrating the effects of CoPP (5 μM, pretreated for 24 h) and pNaKtide (1 μM, pretreated for 1 h) on TCB (100 nM)-induced procollagen-1 expression (**A**, n = 6–8), HO-1 expression (**B**, n = 6–8), c-Src activation (**C**, n = 6–8), ERK1/2 activation (**D**, n = 6–8), and protein carbonylation (**E**, n = 6). Procollagen-1 and HO-1 were determined after 24 h of TCB treatment whereas c-Src activation, ERK1/2 activation, and protein carbonylation were assessed after 1 h of TCB treatment. c-Src activation was expressed as pY418 c-Src/total c-Src (p-Src/t-Src) ratio, and ERK1/2 activation was expressed as phosphor-ERK/total ERK (p-ERK/t-ERK) ratio. For protein carbonylation assay, the Ponceau S stained membrane was used for loading control. *p < 0.05 and **p < 0.01 *vs.* control; ^$^*p* < 0.05 and ^$$^*p* < 0.01 *vs.* TCB alone.

TCB is a cardiotonic steroid. We found the TCB (100 nM, 24 h) induced increases in type I procollagen (procollagen-1) expression (Fig. 1A, *p* < 0.01 *vs.* control). Induction of HO-1 with CoPP (5 μM, 24 h) and inhibition of Na/K-ATPase signaling with pNaKtide (1 μM, 1 h) did not significantly affect procollagen-1 expression at baseline but significantly ameliorated TCB induced increases in procollagen-1 expression (Fig. 1A, both *p* < 0.01 *vs.* TCB alone). Although TCB treatment increased HO-1 expression, the effects of CoPP on HO-1 induction were considerably greater (Fig. 1B). The administration of pNaKtide had a small effect on HO-1 expression which did not attain statistical significance and appeared to be substantially less than that achieved by CoPP (Fig. 1B). TCB also induced activation of c-Src (Fig. 1C) and ERK1/2 (Fig. 1D) as well as oxidant stress as assessed by protein carbonylation (Fig. 1E); these measurements were also attenuated by pretreatment with either CoPP or pNaKtide.

### Effect of pNaKtide and CoPP on PNx-mediated cardiac dysfunction and hemodynamic changes

As this model of experimental renal failure does not induce significant increases in blood pressure (BP) in this mouse strain^16^,^17^, BP measurements were reported only in the Supplementary Materials (details in Supplementary Materials, Table S1). Neither PNx, CoPP nor pNaKtide appeared to have substantial effects on BP in this experiment. Moreover, although PNx was associated with impaired renal function, neither CoPP nor pNaKtide resulted in substantial changes in either plasma cystatin C, creatinine, or urea nitrogen in the setting of PNx (Supplemental Materials, Fig. S1).

**Figure 2:**
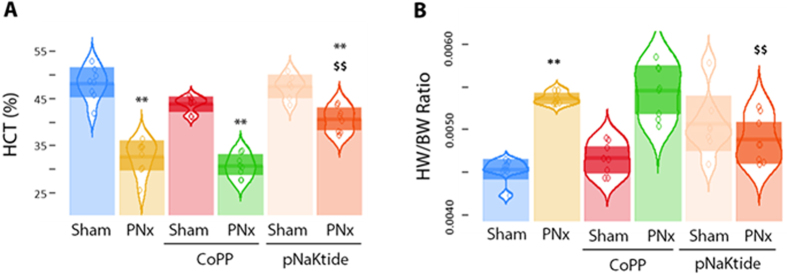
Effect of pNaKtide and CoPP on PNx-induced anemia and heart weight. (**A**) PNx induced anemia was substantially alleviated by administration of pNaKtide but not CoPP (n = 7−8 per group). (**B**) PNx induced increase in heart/body weight ratio was significantly attenuated by administration of pNaKtide (n = 7−8 per group). **p < 0.01, *vs.* Sham; ^$$^*p* < 0.01 *vs.* PNx.

PNx resulted in the consistent development of cardiac hypertrophy and diastolic dysfunction as assessed by echocardiographic methods, summarized in Table 1. Specifically, PNx increased the ventricular wall thickness and mass (anterior wall thickness (AWT), posterior wall thickness (PWT), relative wall thickness (RWT), and left ventricular mass index (LVMI)) as well as the myocardial performance index (MPI). These increases were significantly attenuated by either pNaKtide or CoPP treatment (Table 1). Neither pNaKtide nor CoPP had significant effects as measured by echocardiography in sham treated mice (Table 1). Interestingly, PNx induced profound anemia which was substantially alleviated by concomitant administration of pNaKtide, but not CoPP (Fig. 2A). The increase in heart weight/body weight ratio with PNx was also markedly attenuated by pNaKtide but not CoPP (Fig. 2B).

### Effect of pNaKtide and CoPP on PNx-induced cardiac fibrosis

**Figure 3:**
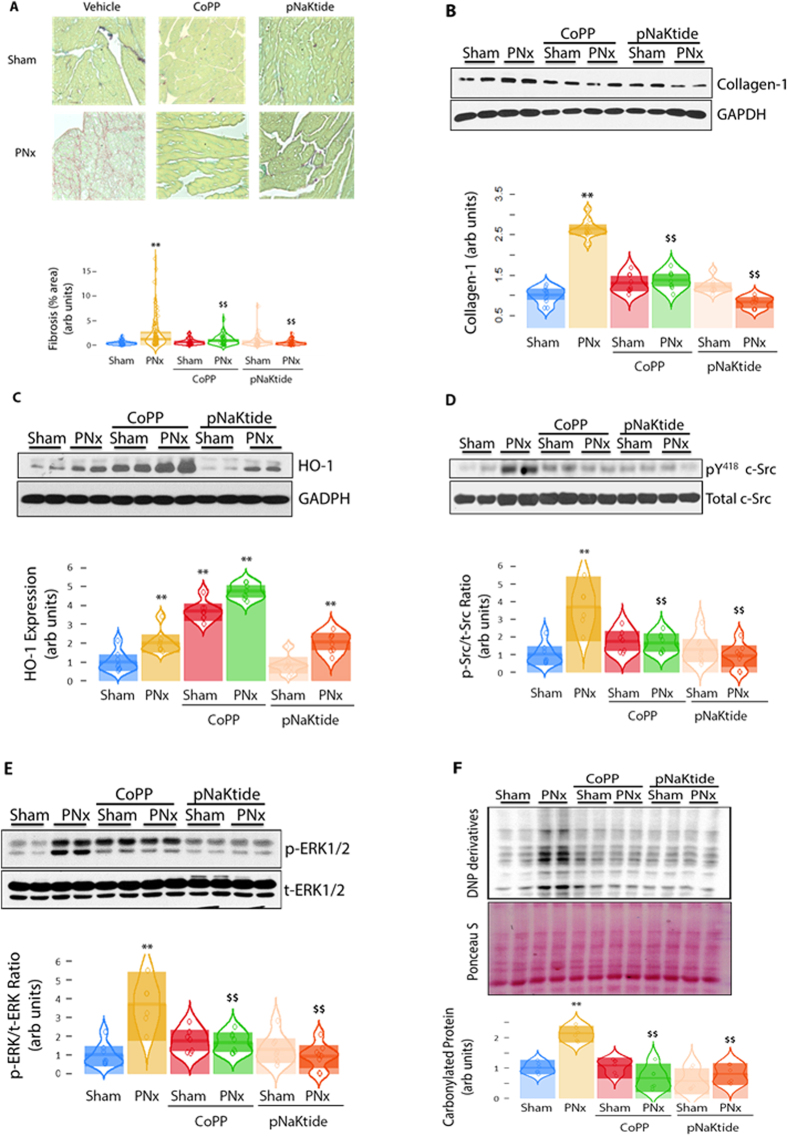
Effect of pNaKtide and CoPP on PNx-induced cardiac fibrosis. (**A**) Representative images of Sirius Red cardiac histology (FAST Green staining as counterstaining), and data analyzed with image J and quantified for Sirius Red staining. For histology analysis in (**A**), 5 spots per section (3 sections X 5 slides per sample) were randomly selected and subjected to analysis using the thresholding function in Image J. (**B**) Representative western blot and data analysis of collagen-1 expression on left ventricle (LV) homogenates, n = 10 mice per group. (**C**) Representative Western blot and data analysis of HO-1 expression on LV homogenates (n = 6–8 mice per group). (**D**) c-Src activation (expressed as pY418 c-Src/total c-Src ratio) determined with western blot on LV homogenates (n = 10 mice per group). (**E**) ERK1/2 activation (expressed as phosphor-ERK/total ERK ratio) determined with Western blot on LV homogenates (n = 6–8 mice per group). (**F**) Representative Western blot analysis of protein carbonylation in LV homogenates with quantitative data (n = 6–8 mice per group). **p < 0.01 *vs.* Sham alone; ^$$^*p* < 0.01 *vs.* PNx alone.

Administration of either pNaKtide or CoPP to sham surgery treated animals did not significantly affect the degree of cardiac fibrosis. PNx surgery was accompanied by marked degrees of cardiac fibrosis as assessed by Sirius Red/Fast Green staining (*p* < 0.01 *vs.* Sham alone) which was significantly attenuated by pNaKtide or CoPP treatment (Fig. 3A, both *p* < 0.01 *vs.* PNx alone).

In addition to the morphological changes, PNx significantly increased collagen-1 expression in left ventricle (LV) homogenates assayed by Western blot (Fig. 3B, *p* < 0.01 *vs.* Sham alone). Administration of either pNaKtide or CoPP reduced PNx-induced increases in LV collagen-1 expression (Fig. 3B, both *p* < 0.01 *vs.* PNx). While CoPP induced HO-1 expression most profoundly, PNx alone also induced HO-1 expression in the LV homogenates (Fig. 3C, *p* < 0.01 *vs.* PNx).

At 4 weeks after PNx surgery, a significant activation of c-Src (Fig. 3D, *p* < 0.01 *vs.* Sham alone) and ERK1/2 (Fig. 3E, *p* < 0.01 *vs.* Sham alone) was observed in LV homogenates in the PNx group that was attenuated in those PNx animals treated with CoPP or pNaKtide (Fig. 3D,E, both *p* < 0.01 *vs.* PNx alone). Compared to the sham group, PNx stimulated protein carbonylation, an oxidative stress marker, in LV homogenates (Fig. 3F, *p* < 0.01 *vs.* Sham alone). Administration of either CoPP or pNaKtide significantly reduced PNx-induced protein carbonylation (Fig. 2F, both *p* < 0.01 *vs.* PNx alone). Lipid peroxidation data assessed by Thiobarbituric Acid Reactive Substances (TBARS) of LV homogenates were consistent with the carbonylation data as expected (Supplemental Materials, Fig. S2).

### Administration of pNaKtide reversed PNx induced uremic cardiomyopathy-reversal study

**Figure 4:**
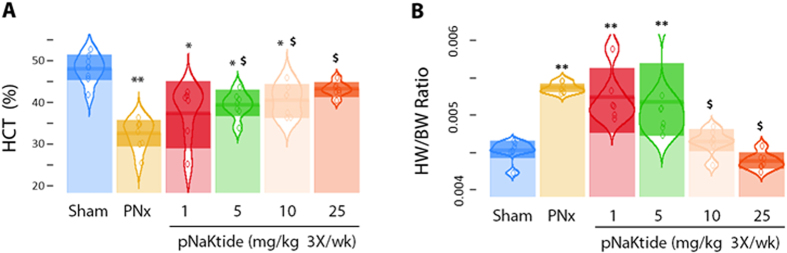
pNaKtide reversed PNx-induced anemia and heart weight/body weight ratio. (**A**) PNx induced anemia was substantially alleviated by administration of pNaKtide (n = 7–8 per group). (**B**) PNx induced increase in heart/body weight ratio was significantly attenuated by administration of pNaKtide (n = 7–8 per group). *p < 0.05 and **p < 0.01, *vs.* Sham; ^$^*p* < 0.05 *vs.* PNx.

**Figure 5:**
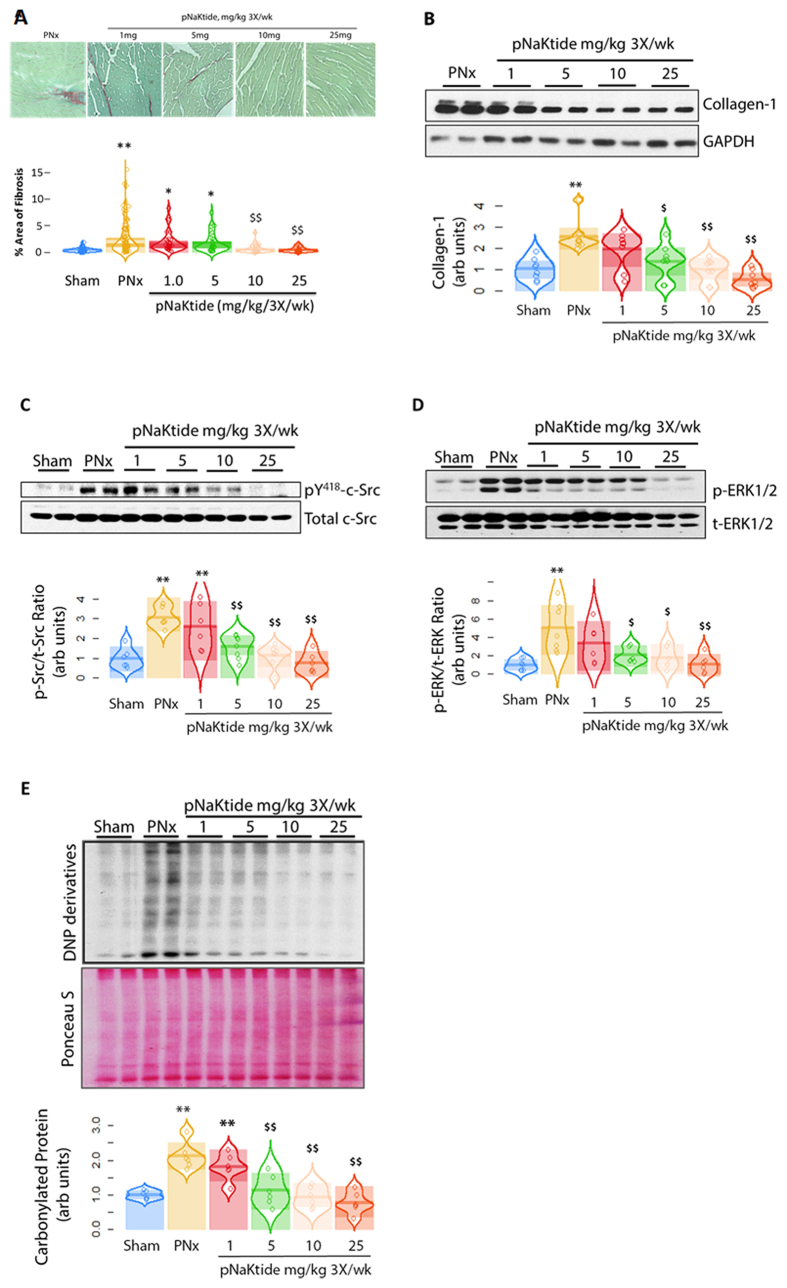
Effect of pNaKtide on reversal of cardiac fibrosis following PNx. (**A**) Representative Sirius red images with quantitative data in PNx animals given vehicle or pNaKtide IP every other day beginning at 4 weeks post-PNx surgery or pNaKtide at 1, 5, 10 and 25 mg/kg with quantitative data (n = 6–12 mice per group). (**B**) Representative Western blots analysis of collagen-1 expression in left ventricle samples with quantitative data. (**C**) Representative phosphorylated c-Src (pY418) and total c-Src Western blots with quantitative data (n = 6–8 mice per group). (**D**) Representative phosphorylated ERK1/2 and total ERK1/2 Western blots with quantitative data (n = 6–8 mice per group). (**E**) Representative Western blot analysis of protein carbonylation in left ventricle samples with quantitative data (n = 6–8 mice per group) **p < 0.01 *vs.* Sham alone; ^$^*p* < 0.05, ^$$^*p* < 0.01 *vs.* PNx vehicle alone.

In another set of animals, PNx was performed and mice were allowed to develop uremic cardiomyopathy for 4 weeks. During the fifth week post PNx, pNaKtide was administered intraperitoneally at a dose of either 0, 1, 5, 10 or 25 mg/kg body weight on day 0, day 2, and day 4, and the mice were sacrificed on day 7 of that week. Blood pressure measurement and echocardiography were performed both before pNaKtide treatment and sacrifice. Again, BP data were not different, and we report these data in the supplement (Supplementary Materials, Table S2). We did note that pNaKtide appeared to reverse anemia and cardiac hypertrophy based on heart weight/body weight ratio, the latter in a dose dependent manner (Fig. 4A,B, respectively). In these animals, we observed that many (but not all) of the echocardiographic features of uremic cardiomyopathy were reversed by pNaKtide in a dose dependent fashion after one week (Table 2). Specifically, left ventricular wall thickness (anterior, posterior and relative wall thickness) as well as left ventricular mass index (LVMI) were ameliorated by pNaKtide at the higher doses. The myocardial performance index (MPI) changes, which were also ameliorated by pNaKtide in the earlier study, were not reversed by pNaKtide in this reversal study. These data are summarized in Table 2. The administration of pNaKtide at higher doses also reversed the fibrosis in a dose dependent manner as assessed by histology (Fig. 5A) and collagen-1 expression (Fig. 5B). Treatment with pNaKtide also attenuated cardiac c-Src activation (Fig. 5C) and ERK1/2 activation (Fig. 5D) as well as oxidant stress as assessed by protein carbonylation (Fig. 5E). Administration of higher doses (10 and 25 mg/kg for plasma creatinine or 25 mg/kg for BUN, respectively) of pNaKtide reversed PNx-mediated increases in plasma creatinine and BUN, but not plasma cystatin C (Supplementary Materials, Fig. S3).

## Discussion

Systemic oxidant stress is certainly part of the uremic syndrome^18^, and plays a critical role in the pathogenesis of the cardiac abnormalities seen with the syndrome called “uremic cardiomyopathy”^19^,^20^,^21^. We have previously demonstrated oxidant stress in experimental uremic cardiomyopathy, a phenomenon which we have attributed to elevated levels of cardiotonic steroids (CTS) which serve as ligands and activators for the Na/K-ATPase^22^,^23^. To this point, we have seen that antagonism of these CTS through active or passive immunization and through pharmacological strategies is effective at ameliorating physiological, morphological, and biochemical features of uremic cardiomyopathy in rodents^24^,^25^,^26^. On this background, we sought to examine whether blockade of Na/K-ATPase oxidant amplification with pNaKtide, an agent which does not affect the pumping function of the Na/K-ATPase, could also effectively ameliorate the phenotypical features of uremic cardiomyopathy. We used CoPP induction of HO-1 to examine whether this was dependent on the specific blockade of Na/K-ATPase oxidant amplification or whether it was a consequence of attenuating the oxidant stress itself.

Our data clearly showed that either pNaKtide or CoPP ameliorated the physiological, morphological, and biochemical alterations of uremic cardiomyopathy. Specifically, the improvement of oxidant stress with either of these two agents resulted in improved left ventricular diastolic function and decreased hypertrophy, less cardiac fibrosis, and less evidence for Na/K-ATPase signaling and ROS stress. Anemia in patients with chronic kidney disease is associated with poor outcome, increased cardiovascular disease and mortality^27^. Surprisingly, amelioration of the anemia associated with chronic renal failure was noted with pNaKtide but not CoPP. We expect this may be related to different effect durations these agents might have in different tissues, but additional work to understand this is critical. In the murine cardiac fibroblast system, we noted that decreasing oxidant stress with either pNaKtide or CoPP attenuated Na/K-ATPase signaling and collagen production to comparable degrees.

We further examined the effects of pNaKtide on established cardiac changes in this model. We found that pNaKtide reversed cardiac hypertrophy and fibrosis in a dose-dependent manner. Interestingly, changes in MPI with PNx, an index of systolic and diastolic function^28^ which were prevented by weekly administration of pNaKtide were not affected by the week of pNaKtide therapy in animals with established uremic cardiomyopathy at any dose studied. This either indicates that the functional change(s) measured with MPI are recalcitrant to reversal, or pNaKtide was administered for inadequate time. Again surprisingly, we found that one week of pNaKtide administration significantly improved established anemia in our model of experimental renal failure.

We believe these data are of interest for several reasons. First, it suggests a somewhat different interpretation of the “chicken or the egg” argument regarding oxidant stress and inflammation. While some excellent work suggests that the oxidant stress in chronic kidney disease results from inflammation presumably due to various uremic toxins^18^, our data suggest that signaling through the Na/K-ATPase may actually produce the initial oxidant stress which initiates recruitment of inflammation. Second and probably more importantly, our data suggest that therapy that allows for the attenuation of oxidant stress can ameliorate phenotypical features of uremic cardiomyopathy. As cardiac mortality is markedly elevated in patients afflicted with advanced renal disease^29^, this suggests the ultimate possibility of effective therapy. We note that neither HO-1 induction nor pNaKtide represent oxygen radical scavengers. Both strategies involve dynamic attenuation of oxidant stress by decreased production and/or increased detoxification. As the clinical administration of reactive oxygen scavengers has been largely disappointing^30^, this distinction is not trivial.

## Materials and Methods

### Experimental design

Male C57BL/6 mice (10–12 weeks old) were purchased from Hilltop Lab Animals Inc. (Scottsdale, PA) and housed in pathogen free animal facility in designated rooms equipped with cages that supply purified air under a 12-hour light/dark cycle. Food and water were supplied *ad libitum*. All animal care and experiments were approved by the Marshall University Institutional Animal Care and Use Committee (IACUC) of the in accordance with the National Institutes of Health (NIH) *Guide for the Care and Use of Laboratory Animals*. The number of animals in each group was determined by power analysis following assumptions derived from our previous study with mice^22^, using a variance of 0.2 within the groups, power of 0.80, and alpha error of 0.05.

1. ***In vivo study I**, **Effects of CoPP and pNaKtide on PNx-induced uremic cardiomyopathy:*** Cobalt protoporphyrin (CoPP, Frontier Scientific, Logan, UT) was used to induce HO-1 expression and pNaKtide was used to block the Na/K-ATPase signaling function. The animals were randomly divided into six groups (10–12 mice per group): (1) Sham surgery (Sham), (2) PNx surgery (PNx), (3) Sham+CoPP (5 mg/kg BW), (4) PNx+CoPP (5 mg/kg BW), (5) Sham+pNaKtide (25 mg/kg BW), and (6) PNx+pNaKtide (25 mg/kg BW).
2. ***In vivo study II**, **Reversal study:*** At week 4 of post-surgery, the animals were randomly divided into different groups and given pNaKtide (0, 1, 5, 10 and 25 mg/kg BW) 3 times (every other day), and then sacrificed 7 days after first injection of pNaKtide.

### 5/6 Nephrectomy Mouse Model

The PNx model utilized a two-step surgical approach. Step-one, instead of partial resection of both poles as reported elsewhere^16^,^22^, the superior and inferior poles of the left kidney were ligated to avoid bleeding, in a manner that approximately only 1/3 of the left kidney mass was left functional. Step-two surgery was operated 7 days later, in which the right kidney was removed. For sham surgery, the two-step surgeries were performed in the same way as in the PNx group, without ligation of the left kidney and removal of right kidney. In the *in vivo* study I, animals were sacrificed 4 weeks after the step-two surgery. In the *in vivo* study II, animals were given either vehicle or pNaKtide beginning at 4 weeks post-surgery for one week and sacrificed at 5 weeks post-surgery.

### Administration of pNaKtide and CoPP

In the *in vivo* study I, pNaKtide was dissolved in sterile PBS buffer and administrated (25 mg/kg BW) by intraperitoneal injection weekly, starting one week after step-two surgery up until the point of sacrificing. CoPP was prepared in Tris-NaOH buffer (25 mM Tris, pH 7.8–8.0) and administrated (5 mg/kg BW) by intraperitoneal injection. CoPP injections were given 5 days prior to, and on the day of, surgery, as well as every 5 days thereafter until sacrificing. In the *in vivo* study II, pNaKtide was administered at 0, 1, 5, 10 and 25 mg/kg 3 times a week beginning at the end of week 4 post-surgery and sacrificed at the end of the additional week.

### Blood Pressure (BP) measurement

Mice were first conditioned in restrainers for at least five days prior to the first BP reading. BP measurements were performed with CODA 8-Channel High Throughput Non-Invasive Blood Pressure System (Kent Scientific, Boston, MA) both one day before step-one surgery, and one day before sacrifice.

### Transthoracic Echocardiography

Transthoracic echocardiography was performed 24 hours before sacrifice. Light anesthesia was achieved by continuous inhalation of isoflurane (1.5–2.5%). Mice in supine position were placed on a heating pad to keep the body temperature at 37 °C. Core body temperature and electrocardiogram (ECG) for physiological monitoring were obtained and manipulated by using VisualSonics Mouse Handling Table (11436) and rectal thermometer. Echocardiographic images were captured using MS400: 18–38 MHz operating frequency MicroScan transducer attached to a Vevo 1100 Imaging System (FUJIFILM VisualSonics Inc.). Warmed echo gel was placed between the probe and shaved chest. B-mode and M-mode images of the heart were obtained from parasternal long axis and short axis. Pulsed-Wave Doppler and Color Doppler were obtained from basal short axis. The average values were calculated from at least four consecutive cardiac cycles. Left-ventricular end-diastolic area (EDA), end-systolic area (ESA), as well as main pulmonary artery diameter was measured from B-mode. End-diastolic diameters (EDD), end-systolic diameters (ESD), as well as anterior and posterior wall thickness (AWT & PWT) were captured from M-mode. Isovolumic contraction and relaxation time (IVCT & IVRT), ejection time (ET), as well as pulmonary velocity time integral (VTI) were obtained from PW and color Doppler. In addition, the following parameters were calculated with the equation below: myocardial performance index (MPI) = (IVCT + IVRT)/ET, relative wall thickness (RWT) = (PWT + AWT)/EDD, cardiac output (CO) = stroke volume (SV) × HR/1000, fractional shortening (FS) = (EDD − ESD)/EDD, ejection fraction (EF) = (EDV − ESV)/EDV and the left ventricular mass index (LVMI) = 1.05[(EDD + PWT + AWT)^3^ − EDD^3^]/Body weight (g).

### Sirius Red/Fast Green staining

Histological staining for collagen fibers was perform on tissues harvested with Sirius Red/Fast Green Collagen Staining solutions (Sigma-Aldrich, St. Louis, MO) according to manufacturer’s protocol. Tissue preparation, fixation, and data analysis were conducted as previously described^25^.

### Western Blotting Analysis

Cardiac (left ventricle, LV) tissue samples and cardiac fibroblast homogenates were prepared and analyzed as we previously described for collagen-1^25^ and other proteins^5^. Assessment of protein carbonylation and activation of c-Src and ERK1/2 was performed as we described in ref. 5. Multiple exposures were analyzed to assure that the signals were within the linear range of the film. The signal density was determined using NIH ImageJ 1.48 v software. Polyclonal antibody against type I collagen was from Southern Biotech (Birmingham, AL). Polyclonal anti-Src [pY418] phospho-specific antibody was from Invitrogen (Camarillo, CA). Monoclonal antibody against total c-Src was from Santa Cruz (Santa Cruz, CA). Antibodies against phospho-ERK1/2 and total ERK1/2 were obtained from Cell Signaling Technology (Danvers, MA). 2,4-dinitrophenylhydrazine (DNPH) and antibody against 2,4-dinitrophenyl (DNP) hydrazone derivatives were from Sigma-Aldrich.

### Experimental design for *in vitro* experiment

C57BL/6 mouse primary cardiac fibroblast cells (purchased from Cell Biologics, Inc., Chicago, IL) were re-suspended in complete fibroblast medium with supplements and 10% FBS (Cell Biologics, Inc.). The cultures were maintained at 37 °C in a 5% CO_2_ incubator and the medium was changed after 48 h and every 2~3 days thereafter. Cardiac fibroblast cells (passage 3–4) were grown until over confluent and were serum-starved (with 1% FBS) overnight before being used for the experiments. Cells were treated with or without TCB (100 nM) to determine collagen-1 and HO-1 expression (24 h treatment), and c-Src and ERK1/2 activation and protein carbonylation (1 h treatment). To determine the effect of HO-1 induction and Na/K-ATPase signaling, some cells were pre-treated with CoPP (5 μM, 24 h) or pNaKtide (1 μM, 1 h) before TCB treatment.

### Measurement of plasma cystatin C, creatinine and blood urea nitrogen (BUN)

The Mouse cystatin C ELISA kit and creatinine kit were obtained from Crystal Chem. Inc. (Downers Grove, IL). The Mouse BUN ELISA kit was obtained from MyBioSource Inc. (San Diego, CA). The measurements were performed following manufactures’ instruction. Each sample was done in duplicate.

### Statistical analysis

Data were tested for normality and then subjected to parametric analysis. When more than two groups were compared, one-way ANOVA was performed prior to comparison of individual groups, and the post-hoc t-tests were adjusted for multiple comparisons using the “Holm” correction. Statistical significance was reported at the *P* < 0.05 and *P* < 0.01 levels. Statistical analyses were performed with the IDE RStudio for the R (version 3.2.5) software (from http://www.rstudio.org and https://www.R-project.org)^31^,^32^. A “pirate” plot was used to form graphs, in which the raw data points, full densities of each group, and Bayesian 95% Highest Density Intervals (HDIs) are plotted^33^ (please see Supplemental Materials, Fig. S5). The HDIs are calculated using the BEST package in R^34^. Values in tables are given as mean ± SEM.

### Data and materials availability

All data needed to evaluate the findings in the paper are present in the paper and the Supplementary Materials.

### Abbreviations used

Although we defined each abbreviation the first time it was mentioned in the manuscript, we have also compiled a table (Table S3, Supplementary Materials) listing these definitions.

## Additional Information

**How to cite this article**: Liu, J. *et al*. Attenuation of Na/K-ATPase Mediated Oxidant Amplification with pNaKtide Ameliorates Experimental Uremic Cardiomyopathy. *Sci. Rep.* **6**, 34592; doi: 10.1038/srep34592 (2016).

## Supplementary Material

Supplementary Information

## Figures and Tables

**Table 1:** Summary of transthoracic echocardiography.

Variable	Sham (n = 18)	PNX (n = 21)	Sham+CoPP (n = 12)	PNX+CoPP (n = 15)	Sham+pNaktide (n = 13)	PNX+pNaktide (n = 14)
BW, g	26.4 ± 0.5	26.1 ± 0.4	28.4 ± 0.6	24.6 ± 0.6	28.1 ± 0.5	25.6 ± 0.6
HR, beat/min	429 ± 5	432 ± 18	425 ± 8	453 ± 11	442 ± 9	414 ± 10
EDA, mm^2^	26.3 ± 0.3	26.3 ± 0.5	28.4 ± 0.5	27.2 ± 0.6	29.6 ± 0.6	27.9 ± 0.5^$^
ESA, mm^2^	17.1 ± 0.3	17.5 ± 0.5	18.3 ± 0.5	19.0 ± 0.1	20.9 ± 0.5	20.5 ± 0.5^$$^
EDD, mm	4.4 ± 0.1	4.4 ± 0.4	4.5 ± 0.04	4.4 ± 0.1	4.6 ± 0.1	4.5 ± 0.5
ESD, mm	3.3 ± 0.1	3.3 ± 0.1	3.4 ± 0.1	3.2 ± 0.1	3.5 ± 0.1	3.4 ± 0.1
PWT, mm	0.59 ± 0.01	0.68 ± 0.01**	0.60 ± 0.01	0.59 ± 0.02^$$^	0.62 ± 0.02	0.59 ± 0.01^$$^
AWT, mm	0.68 ± 0.01	0.77 ± 0.01**	0.67 ± 0.02	0.65 ± 0.01^$$^	0.69 ± 0.01	0.70 ± 0.01^$$^
ET, msec	47 ± 0.6	46 ± 0.8	48 ± 1.0	44 ± 1.0	48 ± 0.8	48 ± 1.1
IVCT+IVRT, (msec)	20 ± 0.5	24 ± 0.7**	19 ± 0.8	21 ± 0.7^$$^	22 ± 0.8	22 ± 0.7^$^
PaVTI	27.52 ± 0.7	27.0 ± 0.5	30.6 ± 1.0	29.9 ± 0.5^$^	28.8 ± 1.1	26.2 ± 0.7
PaD, mm	1.0 ± 0.01	1.0 ± 0.01	1.0 ± 0.02	1.0 ± 0.02	1.0 ± 0.02	1.0 ± 0.07
RWT	0.29 ± 0.01	0.33 ± 0.01**	0.28 ± 0.01	0.28 ± 0.01^$$^	0.29 ± 0.01	0.29 ± 0.01^$$^
MPI	0.43 ± 0.01	0.51 ± 0.01**	0.40 ± 0.01	0.46 ± 0.01^$$^	0.46 ± 0.02	0.46 ± 0.01^$$^
FS, %	25.8 ± 0.8	25.5 ± 1.1	24.7 ± 1.3	26.8. ± 1.7	24.2 ± 1.2	23.4 ± 0.9
EF, %	58.9 ± 1.3	58.1 ± 2.0	57.0 ± 2.2	60.0 ± 2.9	56.1 ± 2.1	54.8 ± 1.5
CO, ml/min	9.59 ± 0.4	9.3 ± 0.3	11.0 ± 0.6	10.5 ± 0.3	10.5 ± 0.3	8.0 ± 0.6
LVMI	3.8 ± 0.1	4.6 ± 0.1**	3.7 ± 0.1	4.0 ± 0.1^$$^	4.0 ± 0.1	4.2 ± 0.1^$^

Values are means ± SE, BW, body weight; HR, heart rate; EDA, end diastolic area; ESA, end systolic area; EDD, end diastolic dimension; ESD, end systolic dimension; PWT, posterior wall thickness; AWT, anterior wall thickness; ET, ejection time; IVCT, isovolumic contraction time; IVRT, isovolumic relaxation time; PaVTI, pulmonary artery velocity time integral; PaD, pulmonary artery dimension; RWT, relative wall thickness; MPI, myocardial performance index; FS, fractional shortening; EF, ejection fraction; CO, cardiac output; LVMI, left ventricle mass index. **p < *0.01 PNx vs. Sham;*^*$*^*p* < *0.05*, ^*$$*^*p* < *0.01* PNx+CoPP or PNx+pNaKtide *vs.* PNx.

**Table 2:** Summary of transthoracic echocardiography.

Variable	PNX vehicle (n = 7)	PNX + 1 mg/kg (n = 6)	PNX + 5 mg/kg (n = 14)	PNX + 10 mg/kg (n = 14)	PNX + 25 mg/kg (n = 7)
BW, g	25.3 ± 1.0	26.8 ± 0.8	25.0 ± 0.8	24.1 ± 0.7	24.3 ± 0.6
HR, beat/min	432 ± 26	389 ± 6	414 ± 9	415 ± 9	392 ± 20
EDA, mm^2^	27.4 ± 0.4	28.0 ± 0.4	27.8 ± 0.6	27.9 ± 0.6	27.5 ± 0.5
ESA, mm^2^	19.4 ± 0.6	20.3 ± 0.3	20.5 ± 0.7	20.6 ± 0.5	20.0 ± 0.4
EDD, mm	4.4 ± 0.1	4.7 ± 0.03	4.6 ± 0.1	4.6 ± 0.1	4.5 ± 0.1
ESD, mm	3.4 ± 0.1	3.6 ± 0.02	3.6 ± 0.1	3.7 ± 0.1	3.6 ± 0.1
PWT, mm	0.62 ± 0.01	0.68 ± 0.02	0.59 ± 0.01^$^	0.60 ± 0.01^$^	0.62 ± 0.0
AWT, mm	0.81 ± 0.03	0.78 ± 0.01	0.68 ± 0.01^$$^	0.64 ± 0.01^$$^	0.63 ± 0.02^$$^
ET, msec	45 ± 1.7	48 ± 1.2	47 ± 1.0	50 ± 1.1	49 ± 2.1
IVCT+IVRT, msec	23 ± 0.7	27 ± 0.7	24 ± 0.8	26 ± 0.7	24 ± 1.6
PaVTI	27.1 ± 0.5	26.6 ± 0.9	26.6 ± 0.8	27.2 ± 0.4	28.2 ± 0.4
PaD, mm	1.1 ± 0.02	1.0 ± 0.02	1.1 ± 0.01	1.0 ± 0.01	1.0 ± 0.02
RWT	0.33 ± 0.01	0.31 ± 0.01	0.27 ± 0.01^$$^	0.27 ± 0.01^$$^	0.28 ± 0.01^$$^
MPI	0.51 ± 0.01	0.55 ± 0.01	0.50 ± 0.01	0.52 ± 0.01	0.49 ± 0.02
FS, %	21.0 ± 1.0	22.7 ± 0.6	21.4 ± 0.8	21.1 ± 0.6	20.9 ± 1.3
EF, %	50.9 ± 2.1	53.8 ± 1.1	51.2 ± 1.5	50.7 ± 1.1	50.2 ± 2.4
CO, ml/min	10.5 ± 1.0	8.3 ± 0.2	9.7 ± 0.5	9.0 ± 0.3	8.8 ± 0.3
LVMI	4.7 ± 0.1	5.1 ± 0.1	4.5 ± 0.1	4.4 ± 0.1^$^	4.3 ± 0.1^$$^

Values are means ± SE, BW, body weight; HR, heart rate; EDA, end diastolic area; ESA, end systolic area; EDD, end diastolic dimension; ESD, end systolic dimension; PWT, posterior wall thickness; AWT, anterior wall thickness; ET, ejection time; IVCT, isovolumic contraction time; IVRT, isovolumic relaxation time; PaVTI, pulmonary artery Velocity time integral; PaD, pulmonary artery dimension; RWT, relative wall thickness; MPI, myocardial performance index; FS, fractional shortening; EF, ejection fraction; CO, cardiac output; LVMI, left ventricle mass index. ^***$***^*p* < *0.05*, ^*$$*^*p* < *0.01 vs.* PNx-vehicle.
