# Corrigendum: Attenuation of Na/K-ATPase Mediated Oxidant Amplification with pNaKtide Ameliorates Experimental Uremic Cardiomyopathy

**DOI:** 10.1038/srep46893

**Published:** 2017-10-20

**Authors:** Jiang Liu, Jiang Tian, Muhammad Chaudhry, Kyle Maxwell, Yanling Yan, Xiaoliang Wang, Preeya T. Shah, Asad A. Khawaja, Rebecca Martin, Tylor J. Robinette, Adee El-Hamdani, Michael W. Dodrill, Komal Sodhi, Christopher A. Drummond, Steven T. Haller, David J. Kennedy, Nader G. Abraham, Zijian Xie, Joseph I. Shapiro

Scientific Reports 6: Article number: 34592; 10.1038/srep34592 published online: 10 04 2016; updated: 10 20 2017.

The Acknowledgements section in this Article is incomplete.

“This work was supported by NIH grants HL109015 (to J.I.S. and Z.X.), HL071556 (to J.I.S.) and HL105649 (to J.T., Z.X. and J.I.S.) as well as HL55601 and HL34300 (to N.G.A.); the Brickstreet Foundation (to J.I.S. and N.G.A.); F32DK104615 (to C.A.D.); AHA 14SDG18650010, The Boone Foundation and an Early Career Development Award from the Central Society for Clinical and Translational Research (to D.J.K.) and the Huntington Foundation Inc.”

should read:

“This work was supported by NIH grants HL109015 (to J.I.S. and Z.X.), DK-106666 (to J.L.), HL071556 (to J.I.S.) and HL105649 (to J.T., Z.X. and J.I.S.) as well as HL55601 and HL34300 (to N.G.A.); the Brickstreet Foundation (to J.I.S. and N.G.A.); F32DK104615 (to C.A.D.); AHA 14SDG18650010, The Boone Foundation and an Early Career Development Award from the Central Society for Clinical and Translational Research (to D.J.K.) and the Huntington Foundation Inc.”

