# Supplementary material for: Attenuation of Na/K-ATPase Mediated Oxidant Amplification with pNaKtide Ameliorates Experimental Uremic Cardiomyopathy
Source: Sci Rep. 2016 Oct 4;6:34592. doi: 10.1038/srep34592 (PMC5048179; doi:10.1038/srep34592)
Supplement: Supplementary Information [file srep34592-s1.pdf]

Attenuation of Na/K-ATPase Mediated Oxidant Amplification with pNaKtide Ameliorates Experimental Uremic Cardiomyopathy

by

Authors: *Jiang Liu[1], *Jiang Tian[2], Muhammad Chaudhry[1], Kyle Maxwell[1], Yanling Yan[1], Xiaoliang Wang[1], Preeya T. Shah[1], Asad A. Khawaja[1], Rebecca Martin[1], Tylor J. Robinette[1], Adee El-Hamdani[1], Michael W. Dodrill[1], Komal Sodhi[1], Christopher A. Drummond[2], Steven T.Haller[2], David J. Kennedy[2], Nader G. Abraham[3,1], Zijian Xie[1], and Joseph I. Shapiro[1$]

From

[1]Marshall University, [2]University of Toledo

and [3]New York Medical College

Corresponding Author:

Joseph I. Shapiro, M.D.

Dean, Joan C. Edwards School of Medicine Professor of Medicine

Marshall University

1600 Medical Center Drive

Huntington, WV 25701-3655

Email: shapiroj@marshall.edu

Phone: 304.691.1700

Fax: 304.691.1726

Results:

Table S1: Blood Pressure Responses to PNx, CoPP and pNaKtide.

Variable	Sham (n=18)	PNx (n=21)	Sham+CoPP (n=12)	PNx+CoPP (n=15)	Sham+pNaktide (n=13)	PNx+pNaktide (n=14)
BASELINE, SBP	114.1±1.7	115.8±2.0	118.0±2.0	119.2±2.5	118.3±2.3	117.9±2.8
BASELINE, DBP	86.3±1.3	89.0±1.4	90.0±1.2	87.4±1.5	89.0±1.6	87.8±1.4
BASELINE, Mean BP	106.0±1.7	105.6±1.4	106.6±0.9	105.9±0.9	102.6±1.3	104.3±1.1
FINAL, SBP	118.2±1.9	119.2±1.5	116.0±1.6	116.1±2.1	118.6±2.1	114.6±1.4
FINAL, DBP	89.2±2.0	91.5±1.8	88.2±1.6	87.1±1.4	87.4±2.1	87.6±1.5
FINAL, Mean BP	107.4±1.8	107.2±3.0	103.8±1.0	105.6±1.2	101.6±2.6	106.7±1.3

BP measurements were performed with CODA 8-Channel High Throughput Non-Invasive Blood Pressure System (Kent Scientific, Boston, MA) both one day before step-one surgery, and one day before sacrifice. Mice were first conditioned in restrainers for at least five days prior to the first baseline and final BP reading. In each group, the baseline represented the baseline BP before sham or PNx surgery, and the final represented the final BP before sacrifice, 4 weeks after sham and PNx surgery (n=12-21 per group). SBP, systolic blood pressure; DBP, diastolic blood pressure; Mean BP, mean arterial pressure. Data presented as mean ± SEM.

Figure S1: CoPP and pNaKtide did not alleviate PNx induced impairment of renal function.

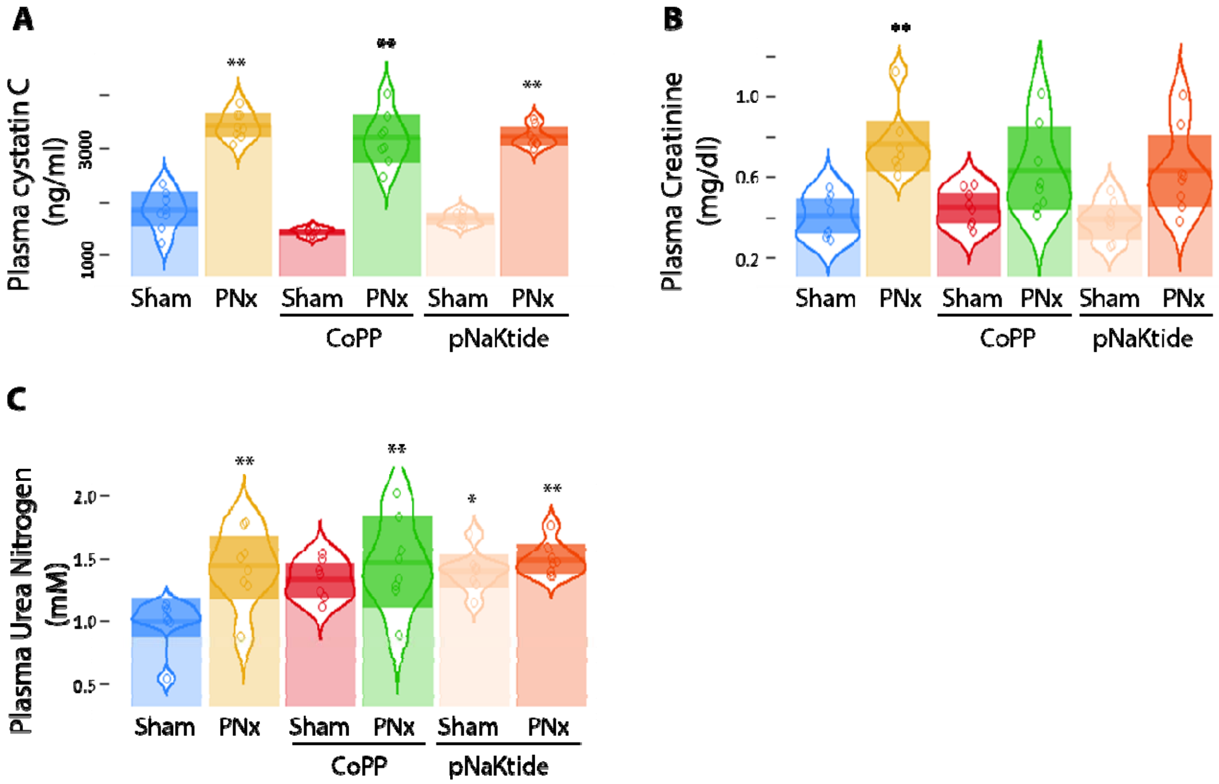

PNx induced impairment of renal function as assessed by plasma cystatin C (**A**), creatinine (**B**), and BUN (**C**), but neither CoPP nor pNaKtide effected significant changes in these measurements (n=7-8 per group). *, $p<0.05$ *vs.* Sham; **, $p<0.01$ *vs.* Sham. The Mouse cystatin C ELISA kit and creatinine kit were obtained from Crystal Chem. Inc. (Downers Grove, IL). The Mouse BUN ELISA kit was obtained from MyBioSource Inc (San Diego, CA). The measurements were performed following manufactures' instruction. Each sample was done in duplicate.

Figure S2: Effect of CoPP and pNaKtide on cardiac oxidant stress:

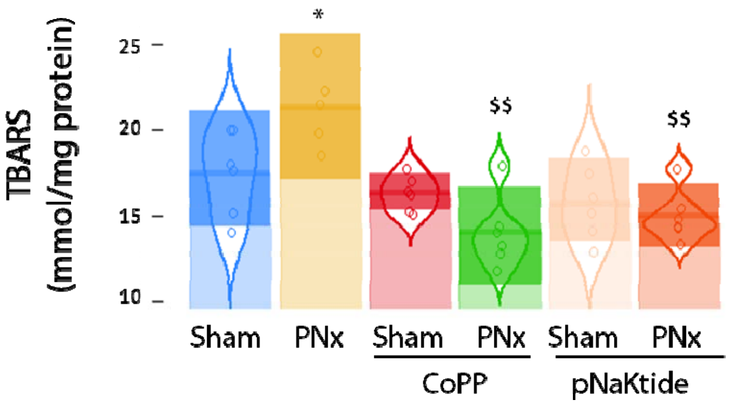

At 4 weeks after PNx surgery, a significant stimulation of lipid peroxidation assayed by Thiobarbituric Acid Reactive Substances (TBARS) was observed in left ventricle homogenates in the PNx group. Lipid peroxidation was attenuated in PNx animals treated with CoPP and pNaKtide (n=7-8 per group). *, p<0.05 *vs.* Sham; $$, p<0.01 *vs*. PNx.

Table S2: ***Effect of pNaKtide on Blood Pressure following 4 weeks after PNx.***

Variable	PNx vehicle (n=21)	PNx+1mg/kg (n=6)	PNx+5mg/kg (n=14)	PNx+10mg/kg (n=14)	PNx+25mg/kg (n=7)
BASELINE, SBP	115.8±2.0	118.2±1.6	120.3±2.3	118.6±2.1	120.9±1.8
BASELINE, DBP	89.0±1.4	88.8±1.4	89.1±1.5	90.1±1.4	89.5±1.8
BASELINE, Mean BP	105.6±1.4	104.0±1.5	102.8±0.7	106.4±1.3	104.4±0.9
FINAL, SBP	119.2±1.5	114.9±2.2	117.7±1.9	117.6±2.0	119.0±1.8
FINAL, DBP	91.5±1.8	85.6±0.4	88.6±1.3	85.3±1.1	87.3±1.3
FINAL, Mean BP	107.2±3.0	94.1±2.0	106.0±1.3	101.1±0.6	103.6±1.1

In the Reversal study, in which pNaKtide was administrated (3x/week for 1 week) to PNx mice 4 weeks after PNx surgery, BP readings were not affected by the different doses of pNaKtide. In each group, the baseline represented the baseline BP obtained 4 weeks after PNx surgery, and the final represented the final BP before sacrifice, obtained after one week of pNaKtide or vehicle injections, thus 5 weeks after sham and PNx surgery (n=6-14 per group). Systolic blood pressure (SBP); diastolic blood pressure (DBP); mean arterial pressure (Mean BP). Data presented as mean ± SEM.

Figure S3: Effect of pNaKtide on renal function in reversal study:

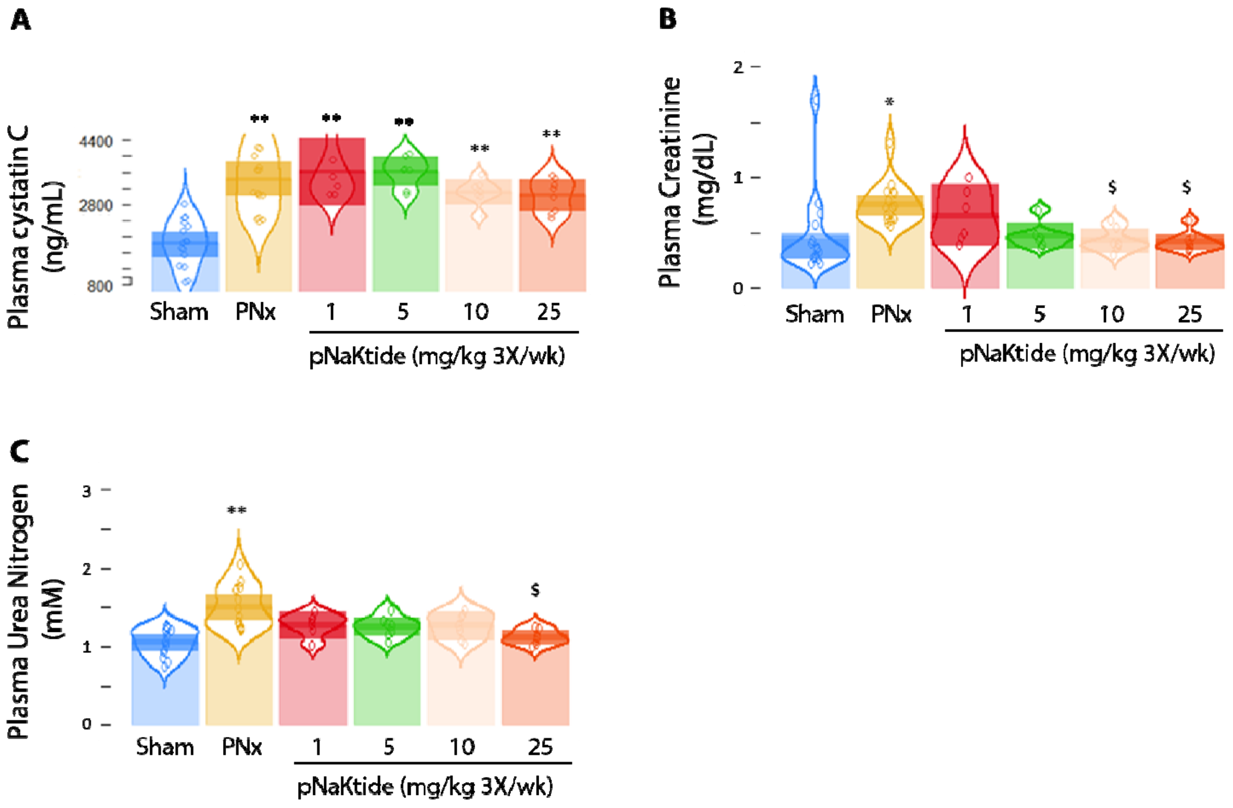

In the Reversal study, pNaKtide was administrated (3x/week for 1 week) to PNx mice 4 weeks after PNx surgery. At higher dose(s), pNaKtide did not reduce PNx-induced cystatin C **(A)**, but pNaKtide reduced PNx-induced high plasma creatinine **(B)** and BUN **(C).** n=6-8 mice per group. ***,** $p<0.05$ *vs.* Sham; ****,** $p<0.01$ *vs.* Sham; $, $p<0.05$ *vs*. PNx.

Figure S4: pNaKtide distribution in mouse cardiac fibroblasts primary culture as well as heart, kidney, and visceral adipose of C57BL/6 mice.

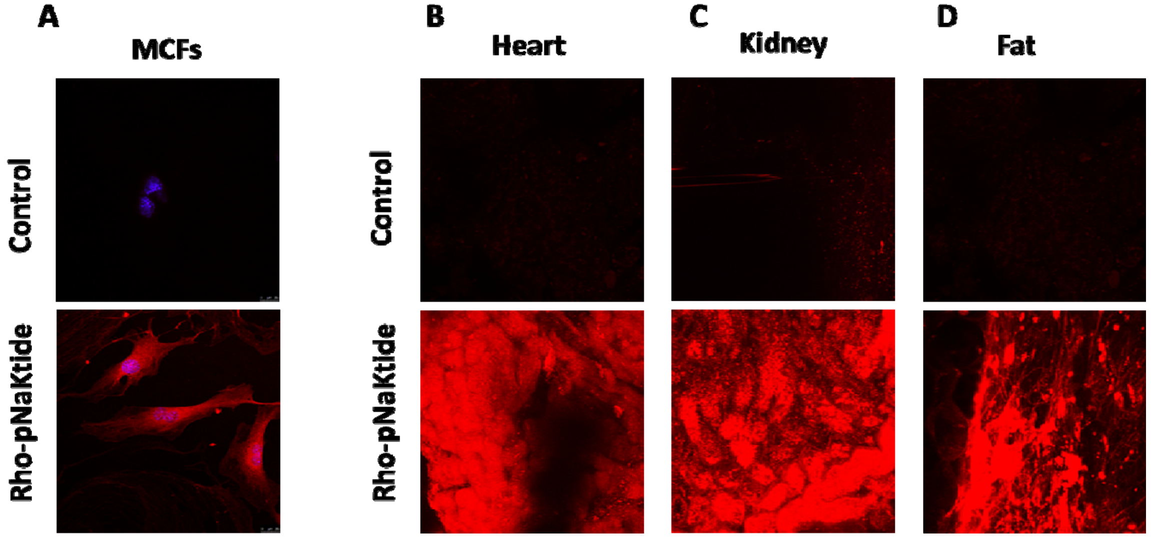

(A) Mouse cardiac fibroblasts were incubated with 1 μM of rhodamine-labeled pNaKtide (Rho-pNaKtide) for 1 hour. Fixed cells were mounted with mounting medium with DAPI (Vector laboratory) (n=4). **(B-D)** Rho-pNaKtide (25mg/kg BW) was administrated to mice by i.p. injection. Vehicle of the same volume was administrated in control mice. After 3 hours, fresh organs (**B** heart, **C** kidney, and **D** visceral adipose) were rinsed with cold 1x PBS, sectioned with surgical blades, and rinsed with cold 1xPBs again. Images were obtained with a 25X water-immerse objective (n=4).

Figure S5. Description of "pirate" plot showing the actual data points as circles, a density distribution of the data as a bean shape or pirate's "beard", the mean as a solid line with the 95% confidence interval for the mean shown as bands about the mean.

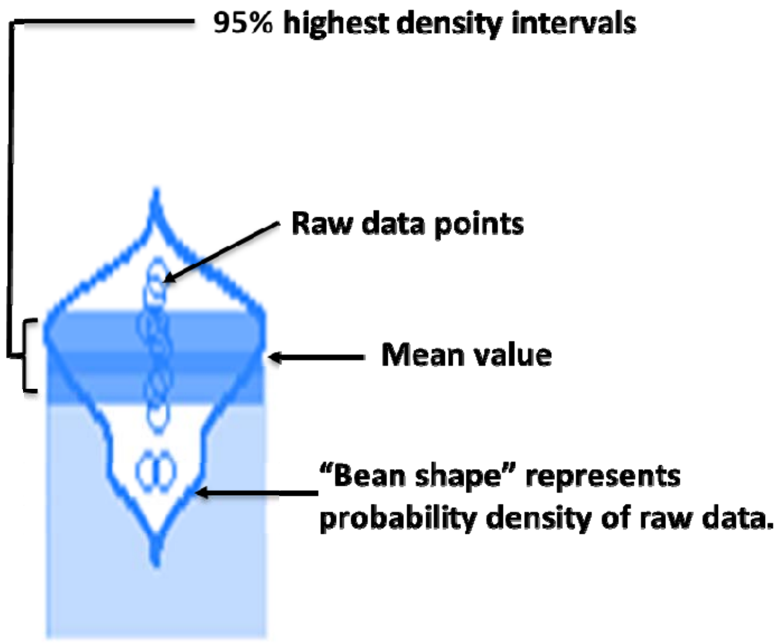

Table S3: ***Abbreviations used.***

AWT,	anterior wall thickness
BP,	blood pressure
BUN,	blood urea nitrogen
CO,	cardiac output
CoPP,	cobalt protoporphyrin
c-Src,	cellular tyrosine-protein kinase Src
CTS,	cardiotonic steroids
ECG,	electrocardiogram
EDA,	left-ventricular end-diastolic area
EDD,	end-diastolic diameter
ERK1/2,	extracellular signal-regulated kinases 1 and 2
ESA,	end-systolic area
ESD,	end-systolic diameter
ET,	ejection time
HO-1,	heme oxygenase-1 (EC 1.14.99.3)
HR,	heart rate
IVCT,	isovolumic contraction
IVRT,	isovolumic relaxation time
LV,	left ventricle
LVMI,	left ventricular mass index
MPI,	myocardial performance index
Na/K-ATPase,	sodium-potassium adenosine triphosphatase (EC 3.6.3.9)
pNaKtide,	a designed peptide derived from the α1 subunit of the Na/K-ATPase
PNx,	5/6 partial nephrectomy
PWT,	posterior wall thickness
ROS,	reactive oxygen species
RWT,	relative wall thickness
TBARS,	thiobarbituric acid reactive substances
TCB,	telecinobufagin, one of cardiotonic steroids
VTI,	pulmonary velocity time integral
